# Supplementary material for: Engulfment by brain macrophages in a short-lived vertebrate
Source: bioRxiv. 2026 Apr 29:2026.04.28.721434. Preprint. [Version 1] doi: 10.64898/2026.04.28.721434 (PMC13142350; doi:10.64898/2026.04.28.721434)
Supplement: Supplement 1 [file NIHPP2026.04.28.721434v1-supplement-1.pdf]

bioRxiv preprint doi: https://doi.org/10.64898/2026.04.28.721434; this version posted April 29, 2026. The copyright holder for this preprint (which was not certified by peer review) is the author/funder, who has granted bioRxiv a license to display the preprint in perpetuity. It is made available under aCC-BY 4.0 International license.

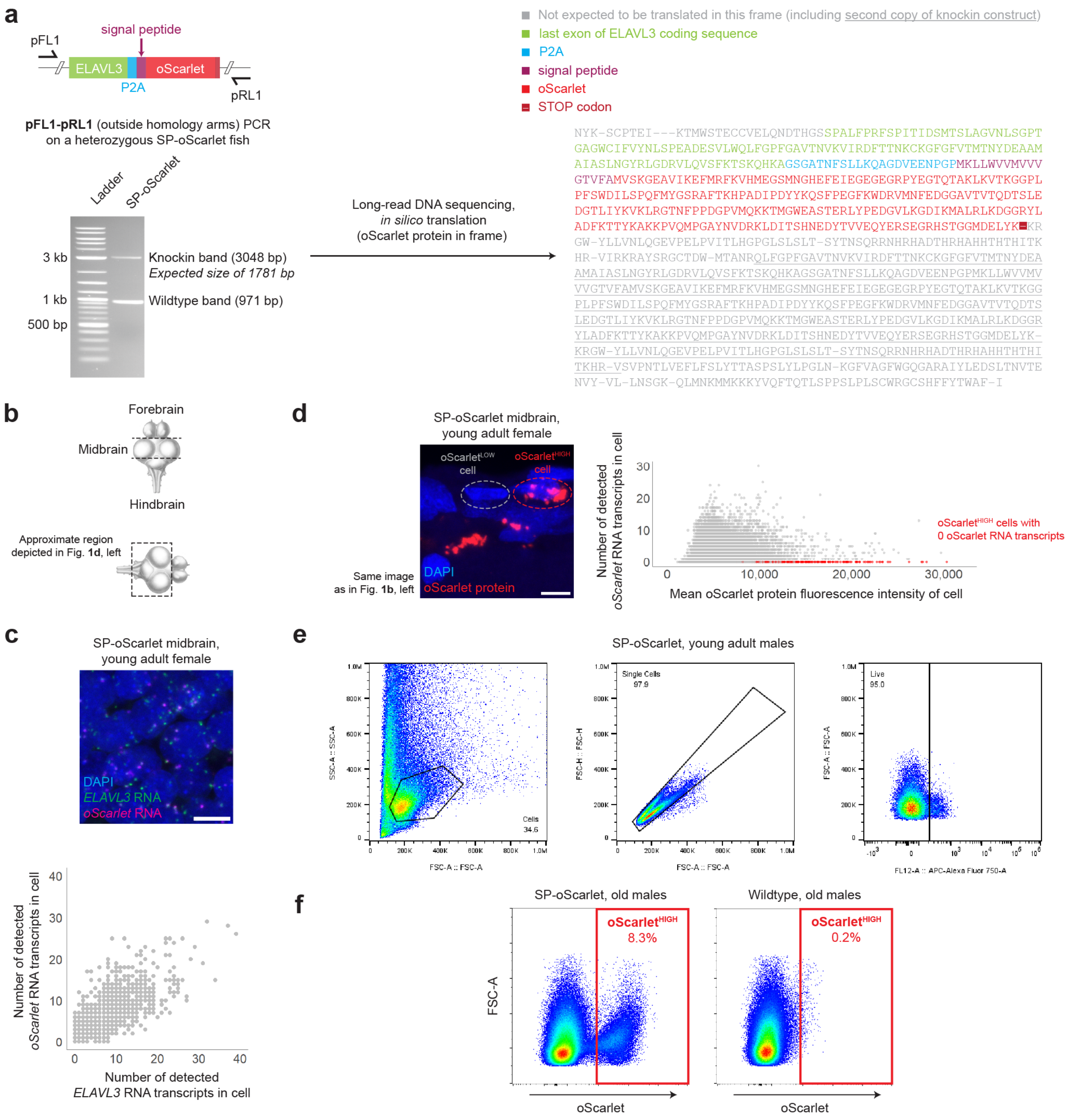

bioRxiv preprint doi: https://doi.org/10.64898/2026.04.28.721434; this version posted April 29, 2026. The copyright holder for this preprint (which was not certified by peer review) is the author/funder, who has granted bioRxiv a license to display the preprint in perpetuity. It is made available under aCC-BY 4.0 International license.

Figure 1 – Figure Supplement 1

a, (Left) Schematic for PCR genotyping of a middle-aged (78 days) male heterozygous SP-oScarlet killifish, and DNA gel result (representative of n = 2 fish, 78 days males, in one experiment).

(Right) *In silico* translated sequence of sequenced SP-oScarlet insertion (n = 1 fish, 78 days male).

b, Representation of killifish brain highlighting approximate brain regions as referred to in this study. Art by Massimo Demma (adapted with permission from D'Angelo et al., 2013[169]).

c, (Above) Representative image of a young adult (59 days) female heterozygous SP-oScarlet killifish brain section, highlighting *ELAVL3* transcripts and *oScarlet* transcripts in the periventricular gray zone, a neuron-dense region of the killifish midbrain. Scale bar = 5 µm.

(Below) Per-cell quantification of number of detected *ELAVL3* transcripts and number of detected *oScarlet* transcripts (1,498 cells from n = 4 fish, 59 days females, with 1 region of interest in 1 sagittal brain section per fish, in one experiment).

d, Same image as in **1b** (left), highlighting examples of oScarlet^{LOW} and oScarlet^{HIGH} cells as detected *in situ*.

(Below) Per-cell quantification of mean oScarlet fluorescence intensity and number of detected *oScarlet* transcripts from experiment in **1c** (25,622 cells from n = 10 fish, 59-63 days females, with 3-6 regions of interest over 1-2 sagittal brain sections per fish, over two experiments). Each dot represents one cell. oScarlet^{HIGH} cells that have 0 detected *oScarlet* RNA transcripts are highlighted in red.

e, FACS plots highlighting gating scheme used to isolate Live cells (as depicted in **1e**) from dissociated young adult male SP-oScarlet brains from experiment in **1d**.

f, FACS plots highlighting oScarlet^{HIGH} cells notably detected in old (130 days old) male heterozygous SP-oScarlet but not in old (122 days old) male wildtype brains (n = 3 fish per genotype, pooled in one experiment). Each dot represents one cell.

bioRxiv preprint doi: https://doi.org/10.64898/2026.04.28.721434; this version posted April 29, 2026. The copyright holder for this preprint (which was not certified by peer review) is the author/funder, who has granted bioRxiv a license to display the preprint in perpetuity. It is made available under aCC-BY 4.0 International license.

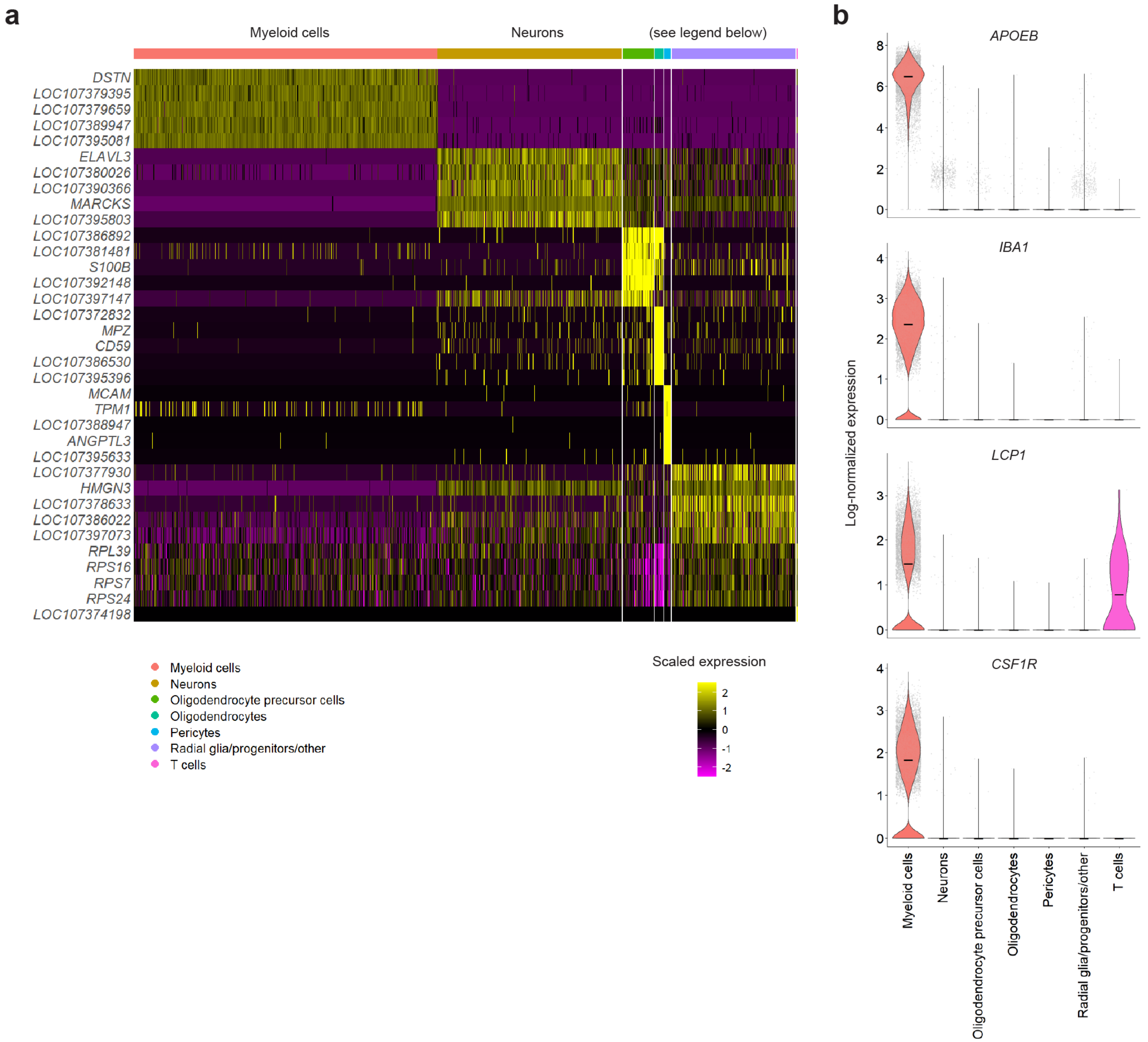

630

bioRxiv preprint doi: https://doi.org/10.64898/2026.04.28.721434; this version posted April 29, 2026. The copyright holder for this preprint (which was not certified by peer review) is the author/funder, who has granted bioRxiv a license to display the preprint in perpetuity. It is made available under aCC-BY 4.0 International license.

Figure 1 – Figure Supplement 2

a, Heatmap of expression of top 5 marker genes per cell type, as ranked by area under curve (Presto), in every young adult cell from experiment in **1d**. Full list of cell type marker genes in **Supplementary Table 8**.

b, Violin plots from experiment in **1d** highlighting expression of *APOEB* (*LOC10737395*), *IBA1* (*LOC107378674*), *LCP1* (*LOC107389591*), and *CSF1R* (*LOC107387189*) in each cell type. Each dot represents one cell. Lines: medians.

bioRxiv preprint doi: https://doi.org/10.64898/2026.04.28.721434; this version posted April 29, 2026. The copyright holder for this preprint (which was not certified by peer review) is the author/funder, who has granted bioRxiv a license to display the preprint in perpetuity. It is made available under aCC-BY 4.0 International license.

a

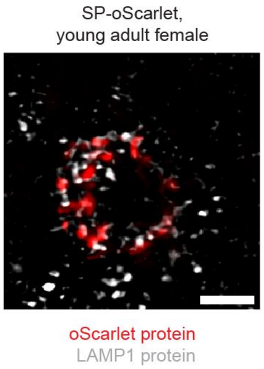

b

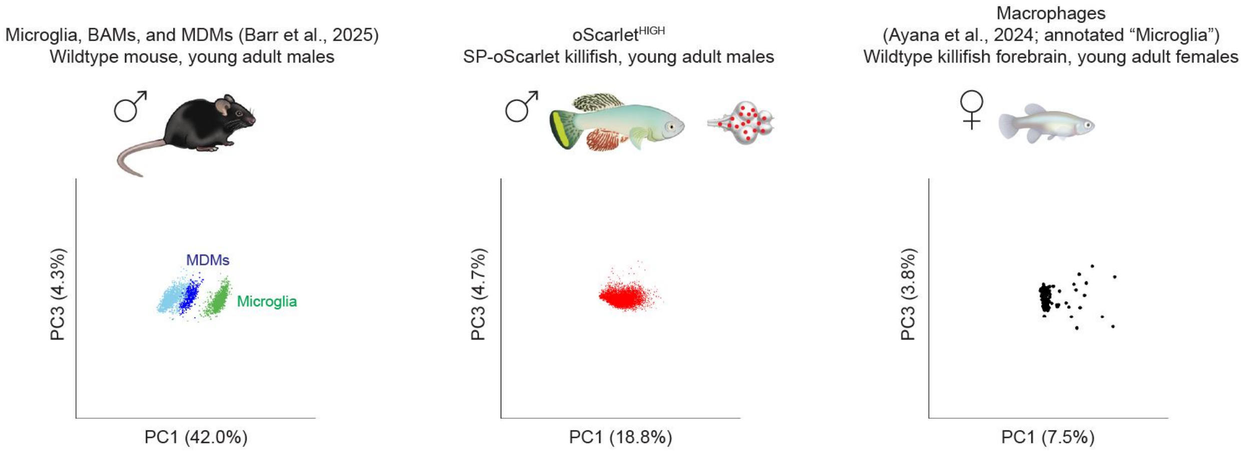

bioRxiv preprint doi: https://doi.org/10.64898/2026.04.28.721434; this version posted April 29, 2026. The copyright holder for this preprint (which was not certified by peer review) is the author/funder, who has granted bioRxiv a license to display the preprint in perpetuity. It is made available under aCC-BY 4.0 International license.

Figure 2 – Figure Supplement 1

a, Representative image of a young adult (59 days) female heterozygous SP-oScarlet killifish brain section, highlighting oScarlet protein signal colocalized with the late endosome and lysosome marker LAMP1 in an SP-oScarlet brain (representative of n = 8 fish, 59-63 days females, over two experiments). Scale bar = 2 μm.

b, Principal component analysis (PCA) plots (principal components 1 vs. 3) of wildtype young adult mouse brain macrophages (Barr et al., 2025[75]), young adult oScarlet^{HIGH} cells from experiment in **1d**, and wildtype young adult killifish forebrain macrophages (Ayana et al., 2024[68]) using marker genes of microglia, BAMs, and MDMs from the Barr et al. dataset, or all killifish homologs of these genes. Each dot represents one cell. Percentages on axes represent the percentage of variance explained by each respective principal component.

bioRxiv preprint doi: https://doi.org/10.64898/2026.04.28.721434; this version posted April 29, 2026. The copyright holder for this preprint (which was not certified by peer review) is the author/funder, who has granted bioRxiv a license to display the preprint in perpetuity. It is made available under aCC-BY 4.0 International license.

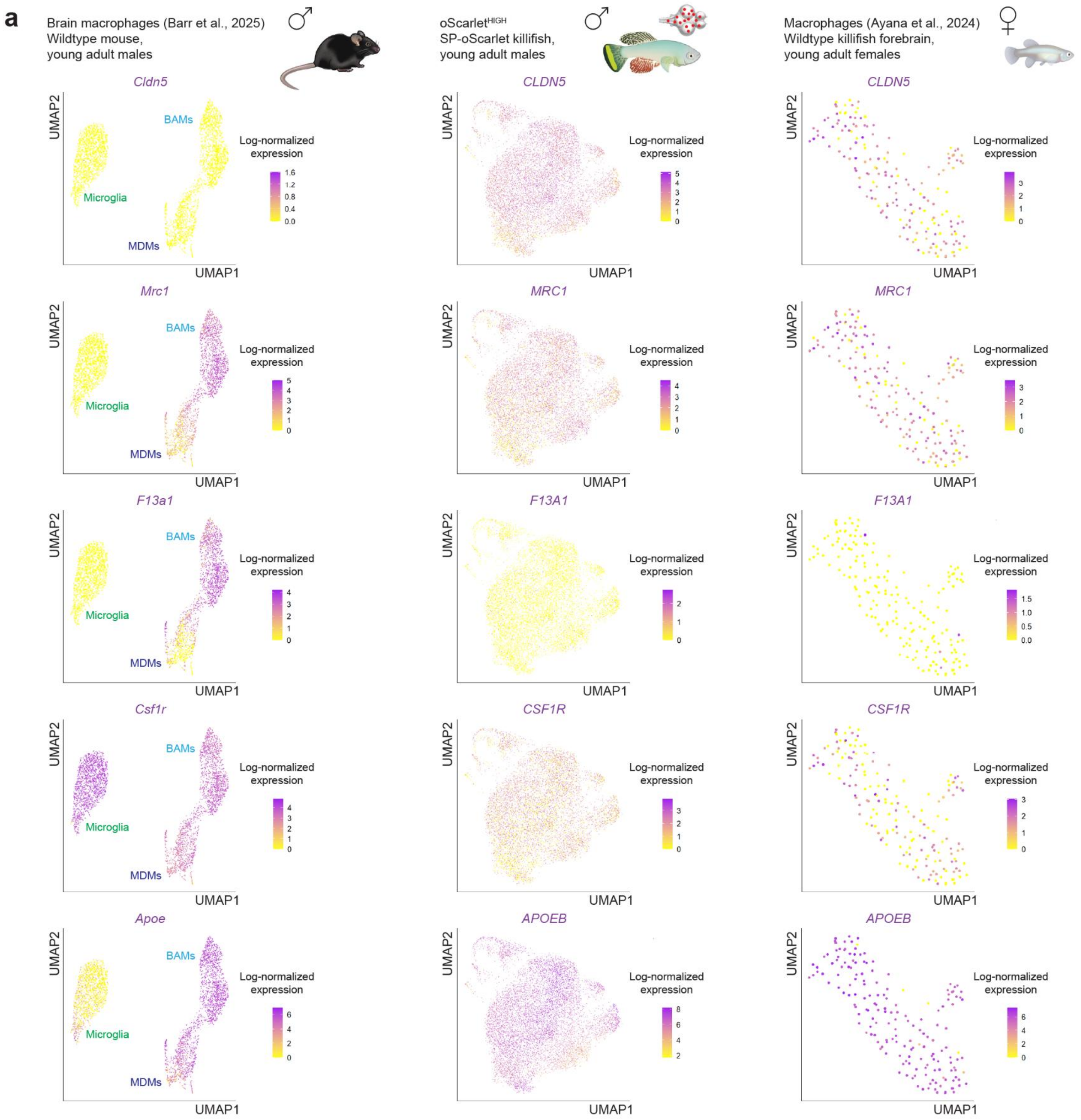

bioRxiv preprint doi: https://doi.org/10.64898/2026.04.28.721434; this version posted April 29, 2026. The copyright holder for this preprint (which was not certified by peer review) is the author/funder, who has granted bioRxiv a license to display the preprint in perpetuity. It is made available under aCC-BY 4.0 International license.

Figure 2 – Figure Supplement 2

a, UMAPs of wildtype young adult mouse brain macrophages (Barr et al., 2025[75]), young adult oScarlet^{HIGH} cells from experiment in **1d**, and wildtype young adult killifish forebrain macrophages (Ayana et al., 2024[68]) colored according to log-normalized *Cldn5* (or killifish homolog *CLDN5* (*LOC107394244*)), *Mrc1* (or killifish homolog *MRC1* (*LOC107383768*)), *F13a1* (or killifish homolog *F13A1* (*LOC107377057*)), *Csf1r* (or killifish homolog *CSF1R* (*LOC107381415*)), or *Apoe* (or killifish homolog *APOEB* (*LOC107379395*)) expression. Each dot represents one cell.

bioRxiv preprint doi: https://doi.org/10.64898/2026.04.28.721434; this version posted April 29, 2026. The copyright holder for this preprint (which was not certified by peer review) is the author/funder, who has granted bioRxiv a license to display the preprint in perpetuity. It is made available under aCC-BY 4.0 International license.

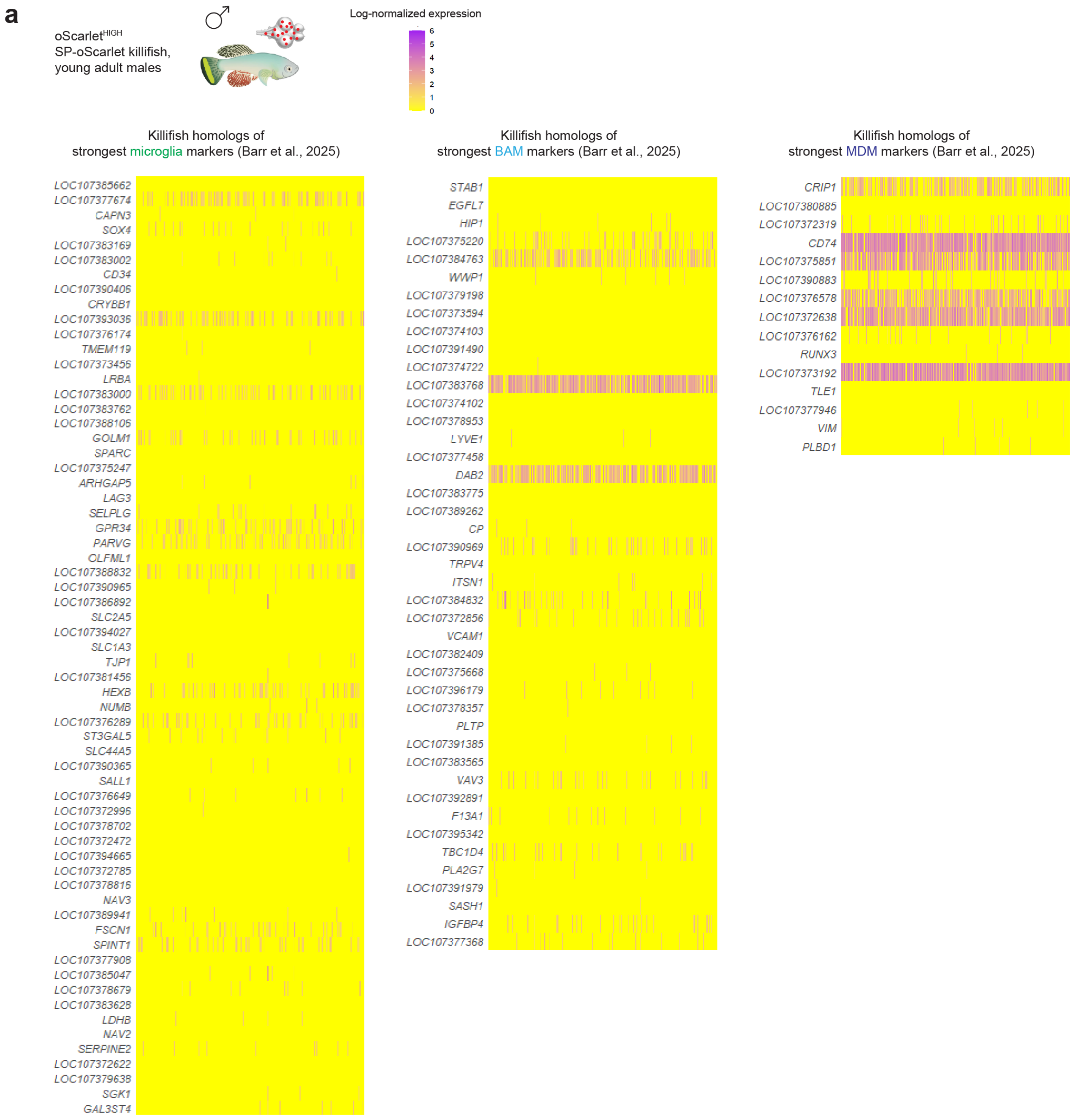

bioRxiv preprint doi: https://doi.org/10.64898/2026.04.28.721434; this version posted April 29, 2026. The copyright holder for this preprint (which was not certified by peer review) is the author/funder, who has granted bioRxiv a license to display the preprint in perpetuity. It is made available under aCC-BY 4.0 International license.

Figure 2 – Figure Supplement 3

a, Heatmaps of expression of killifish homologs of strongest microglia, BAM, and MDM marker genes (Barr et al., 2025[75]) in young adult oScarlet^{HIGH} cells from experiment in **1d**.

bioRxiv preprint doi: https://doi.org/10.64898/2026.04.28.721434; this version posted April 29, 2026. The copyright holder for this preprint (which was not certified by peer review) is the author/funder, who has granted bioRxiv a license to display the preprint in perpetuity. It is made available under aCC-BY 4.0 International license.

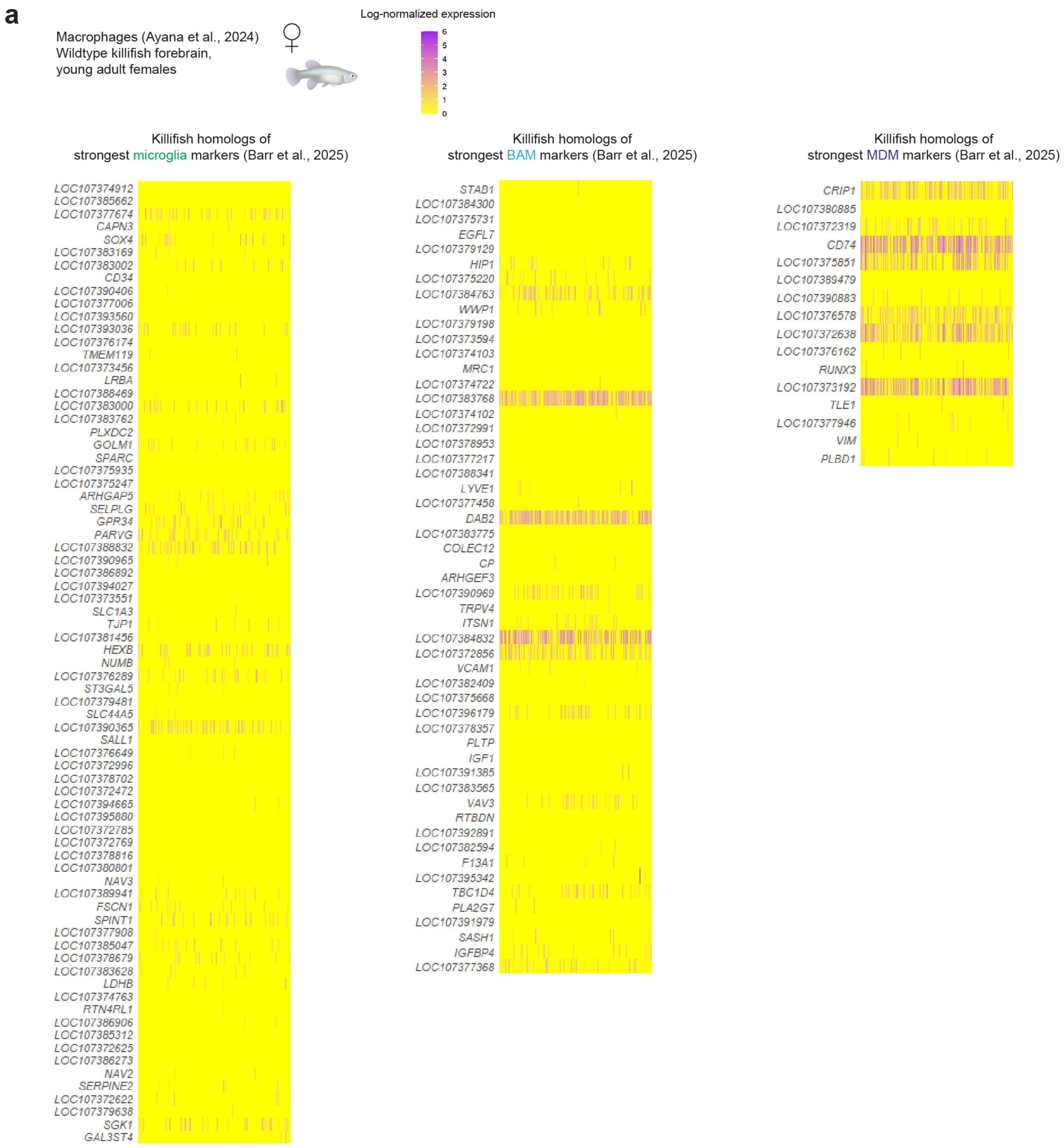

bioRxiv preprint doi: https://doi.org/10.64898/2026.04.28.721434; this version posted April 29, 2026. The copyright holder for this preprint (which was not certified by peer review) is the author/funder, who has granted bioRxiv a license to display the preprint in perpetuity. It is made available under aCC-BY 4.0 International license.

Figure 2 – Figure Supplement 4

a, Heatmaps of expression of killifish homologs of strongest microglia, BAM, and MDM marker genes (Barr et al., 2025[75]) in wildtype young adult killifish forebrain macrophages (Ayana et al., 2024[68]).

bioRxiv preprint doi: https://doi.org/10.64898/2026.04.28.721434; this version posted April 29, 2026. The copyright holder for this preprint (which was not certified by peer review) is the author/funder, who has granted bioRxiv a license to display the preprint in perpetuity. It is made available under aCC-BY 4.0 International license.

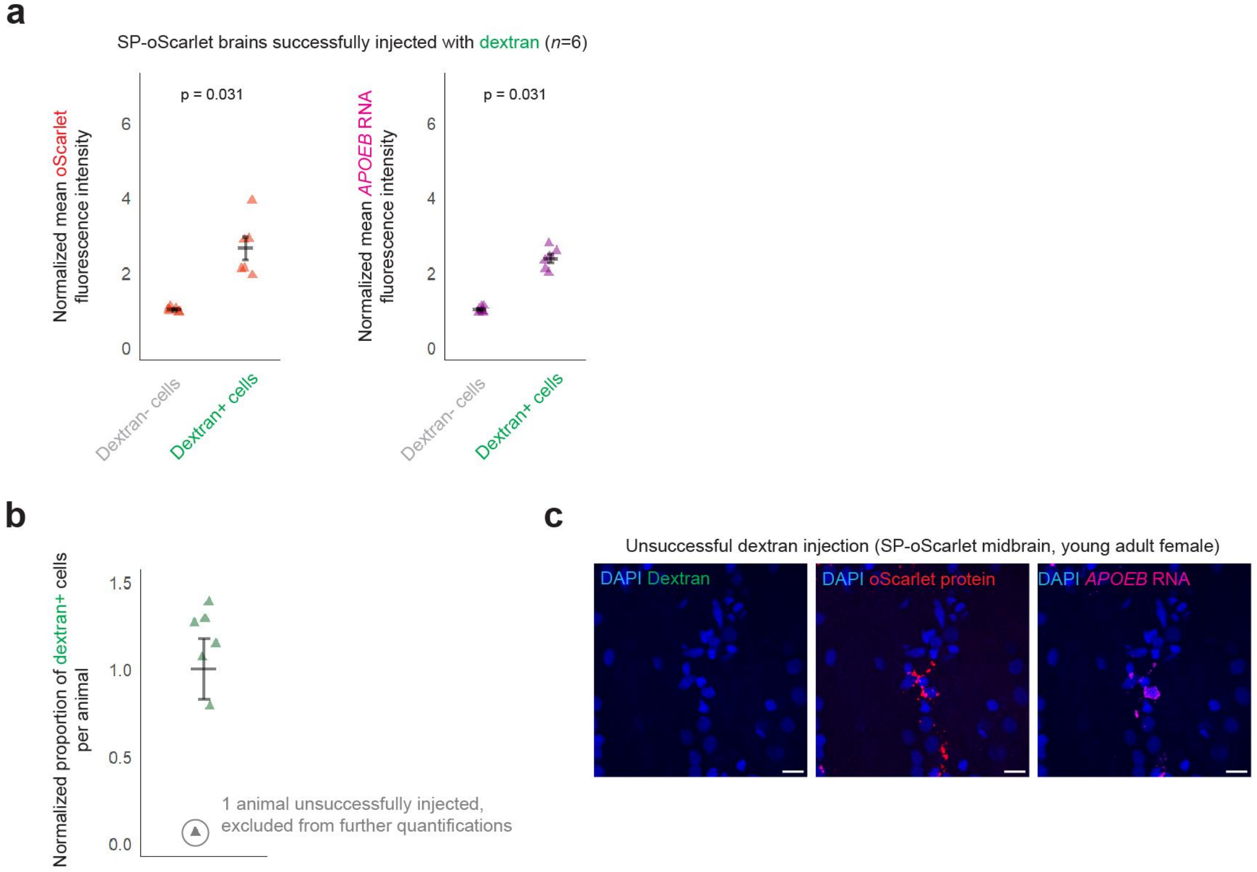

bioRxiv preprint doi: https://doi.org/10.64898/2026.04.28.721434; this version posted April 29, 2026. The copyright holder for this preprint (which was not certified by peer review) is the author/funder, who has granted bioRxiv a license to display the preprint in perpetuity. It is made available under aCC-BY 4.0 International license.

Figure 3 – Figure Supplement 1

a, Quantification of mean oScarlet fluorescence intensity and mean *APOEB* RNA fluorescence intensity in dextran-negative and dextran-positive cells (n = 6 successfully injected fish, 63-91 days females, with 5-6 regions of interest over 1-2 sagittal brain sections per fish, over two experiments). Four images (one image per fish for each of four fish) of regions of interest with a visually apparent very high density of cells with high *APOEB* RNA expression, which could plausibly reflect an injection-related injury, were excluded from quantifications. The excluded images are listed in **Supplementary Table 4**. Each triangle represents one fish. Mean +/- standard error of mean; p-values, paired Wilcoxon rank-sum test at per-animal level.

b, Quantification from experiment in **3a** of proportion of dextran-positive cells per fish (n = 7 fish, 63-91 days females, with 5-6 regions of interest over 1-2 sagittal brain sections per fish, over two experiments), highlighting in grey one fish in which dextran was not observed to circulate throughout the brain (also confirmed by visual inspection of sections). This unsuccessfully injected fish (63 days, female) was excluded from subsequent quantifications. Each triangle represents one fish.

c, Representative image of brain sections from the young adult (63 days) female heterozygous SP-oScarlet killifish unsuccessfully injected with dextran (n = 1 fish). Scale bars = 10 μm.

bioRxiv preprint doi: https://doi.org/10.64898/2026.04.28.721434; this version posted April 29, 2026. The copyright holder for this preprint (which was not certified by peer review) is the author/funder, who has granted bioRxiv a license to display the preprint in perpetuity. It is made available under aCC-BY 4.0 International license.

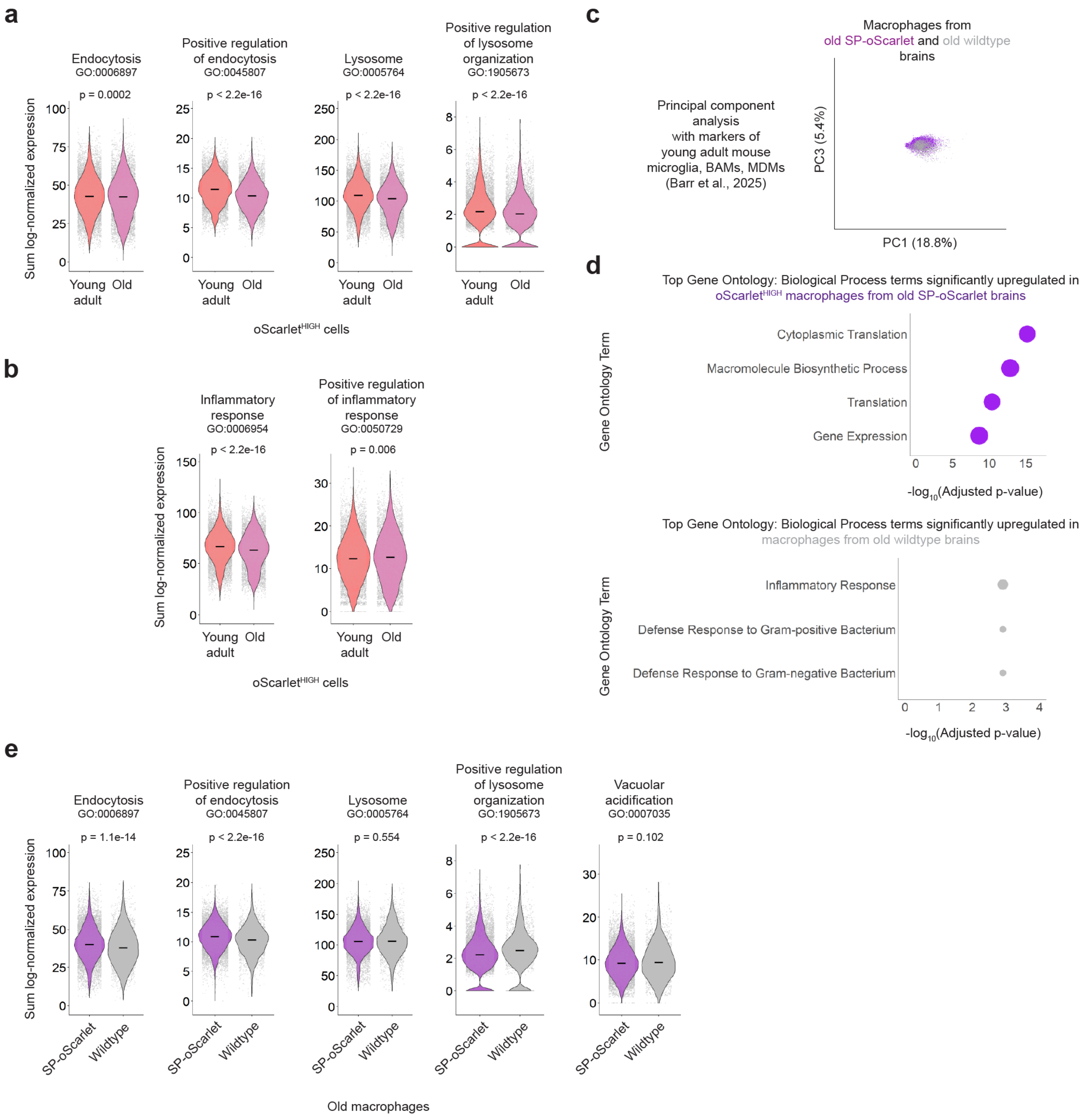

bioRxiv preprint doi: https://doi.org/10.64898/2026.04.28.721434; this version posted April 29, 2026. The copyright holder for this preprint (which was not certified by peer review) is the author/funder, who has granted bioRxiv a license to display the preprint in perpetuity. It is made available under aCC-BY 4.0 International license.

Figure 4 – Figure Supplement 1

a, Violin plots for sum log-normalized expression of genes in Gene Ontology: Biological Process terms “endocytosis” (GO:0006897), “positive regulation of endocytosis” (GO:0045807), “lysosome” (GO:0005764), and “positive regulation of lysosome organization” (GO:1905673) in young adult and old oScarlet^{HIGH} cells from experiment in **1d**. Each dot represents one cell. Lines: medians; p-values, unpaired Wilcoxon rank-sum test at per-cell level (fish were pooled for single cell RNA-sequencing experiments to obtain sufficient numbers of oScarlet^{HIGH} cells).

b, Violin plots for sum log-normalized expression of genes in Gene Ontology: Biological Process terms “inflammatory response” (GO:0006954) and “positive regulation of inflammatory response” (GO:0050729) in young adult and old oScarlet^{HIGH} cells from experiment in **1d**. Each dot represents one cell. Lines: medians; p-values, unpaired Wilcoxon rank-sum test at per-cell level.

c, PCA plot (principal components 1 vs. 3) of oScarlet^{HIGH} and wildtype old killifish brain macrophages from experiment in **4e** using all killifish homologs of marker genes of microglia, BAMs, and MDMs (Barr et al., 2025[75]). Each dot represents one cell. Percentages on axes represent the percentage of variance explained by each respective principal component.

d, Top Gene Ontology: Biological Process terms upregulated in old oScarlet^{HIGH} macrophages (above) and in old wildtype macrophages (below) from experiment in **4e**, as ranked by adjusted p-value (Fisher’s exact test, Benjamini-Hochberg FDR-corrected). Size of each circle corresponds to number of distinct human homologs among differentially expressed genes from each term. Full lists of differentially expressed genes and upregulated terms in **Supplementary Tables 12-14**.

e, Violin plots for sum log-normalized expression of genes in Gene Ontology: Biological Process terms “endocytosis” (GO:0006897), “positive regulation of endocytosis” (GO:0045807), “lysosome” (GO:0005764), “positive regulation of lysosome organization” (GO:1905673), and “vacuolar acidification” (GO: 0007035) in old oScarlet^{HIGH} and old wildtype macrophages from experiment in **4e**. Each dot represents one cell. Lines: medians; p-values, unpaired Wilcoxon rank-sum test at per-cell level.

bioRxiv preprint doi: https://doi.org/10.64898/2026.04.28.721434; this version posted April 29, 2026. The copyright holder for this preprint (which was not certified by peer review) is the author/funder, who has granted bioRxiv a license to display the preprint in perpetuity. It is made available under aCC-BY 4.0 International license.

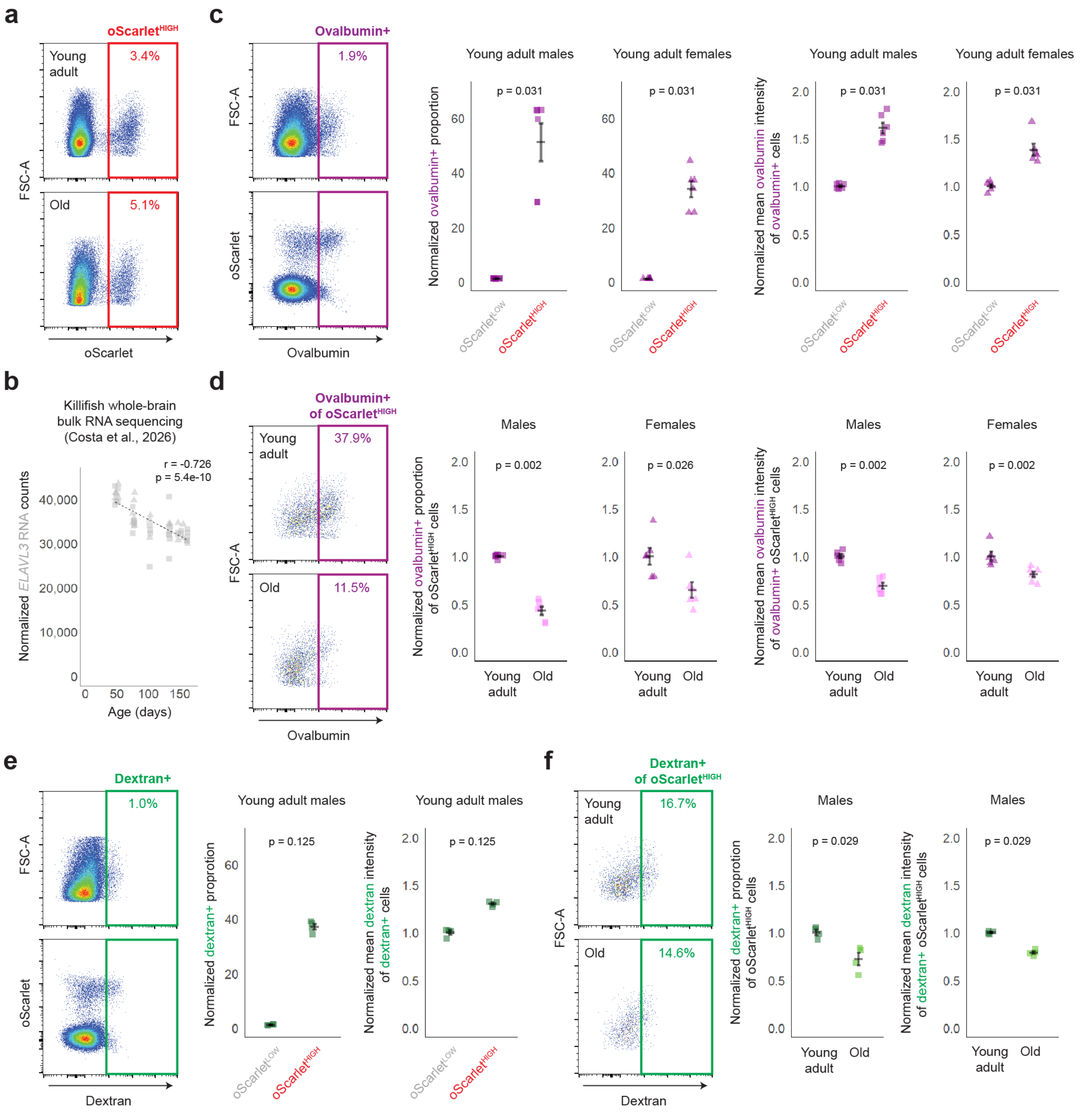

bioRxiv preprint doi: https://doi.org/10.64898/2026.04.28.721434; this version posted April 29, 2026. The copyright holder for this preprint (which was not certified by peer review) is the author/funder, who has granted bioRxiv a license to display the preprint in perpetuity. It is made available under aCC-BY 4.0 International license.

Figure 5 – Figure Supplement 1

a, Representative flow cytometry plots from young adult (41 days, above) and old (160 days, below) male heterozygous SP-oScarlet killifish brains, highlighting oScarlet^{HIGH} cells. Each dot represents one cell.

b, Plot of normalized *ELAVL3* (*LOC107378364*) RNA counts across age from bulk RNA sequencing of whole killifish brains (Costa et al., 2026[55]). Each square (male) or triangle (female) represents one fish. Dashed line: linear regression. r: Pearson correlation coefficient; p-value, two-sided t-test.

c, (Left) Representative flow cytometry plots from young adult (41 days) male heterozygous SP-oScarlet killifish brains, highlighting ovalbumin+ cells as identified from all Live cells, with mean ovalbumin fluorescence intensity plotted against either FSC-A (above) or mean oScarlet fluorescence intensity (below). Each dot represents one cell.

(Right) Quantifications of proportion of ovalbumin+ cells and mean ovalbumin fluorescence intensity of ovalbumin+ cells among young adult oScarlet^{LOW} and oScarlet^{HIGH} cells (same experiments as in **5a**). Each square (male) or triangle (female) represents one fish. Mean +/- standard error of mean; p-values, paired Wilcoxon rank-sum test at per-animal level.

d, (Left) Representative flow cytometry plots from young adult (41 days) (above) and old (160 days) (below) male heterozygous SP-oScarlet killifish brains, highlighting ovalbumin+ oScarlet^{HIGH} cells. Each dot represents one cell.

(Right) Quantifications of proportion of ovalbumin+ cells and mean ovalbumin fluorescence intensity of ovalbumin+ cells among young adult and old oScarlet^{HIGH} cells (same experiments as in **5a**). Each square (male) or triangle (female) represents one fish. Mean +/- standard error of mean; p-values, unpaired Wilcoxon rank-sum test at per-animal level.

e, (Left) Representative flow cytometry plots from young adult (56 days) male heterozygous SP-oScarlet killifish brains, highlighting dextran+ cells as identified from all Live cells, with mean dextran fluorescence intensity plotted against either FSC-A (above) or mean oScarlet fluorescence intensity (below). Each dot represents one cell.

(Right) Quantifications of proportion of dextran+ cells and mean dextran fluorescence intensity of dextran+ cells among young adult oScarlet^{LOW} and oScarlet^{HIGH} cells (same experiment as in **5e**). Each square represents one fish. Mean +/- standard error of mean; p-values, paired Wilcoxon rank-sum test at per-animal level.

f, (Left) Representative flow cytometry plots from young adult (56 days) (above) and old (156 days) (below) male heterozygous SP-oScarlet killifish brains, highlighting dextran+ oScarlet^{HIGH} cells. Each dot represents one cell.

(Right) Quantifications of proportion of dextran+ cells and mean dextran fluorescence intensity of dextran+ cells among young adult and old oScarlet^{HIGH} cells (same experiment as in **5e**). Each square represents one fish. Mean +/- standard error of mean; p-values, unpaired Wilcoxon rank-sum test at per-animal level.

bioRxiv preprint doi: https://doi.org/10.64898/2026.04.28.721434; this version posted April 29, 2026. The copyright holder for this preprint (which was not certified by peer review) is the author/funder, who has granted bioRxiv a license to display the preprint in perpetuity. It is made available under aCC-BY 4.0 International license.

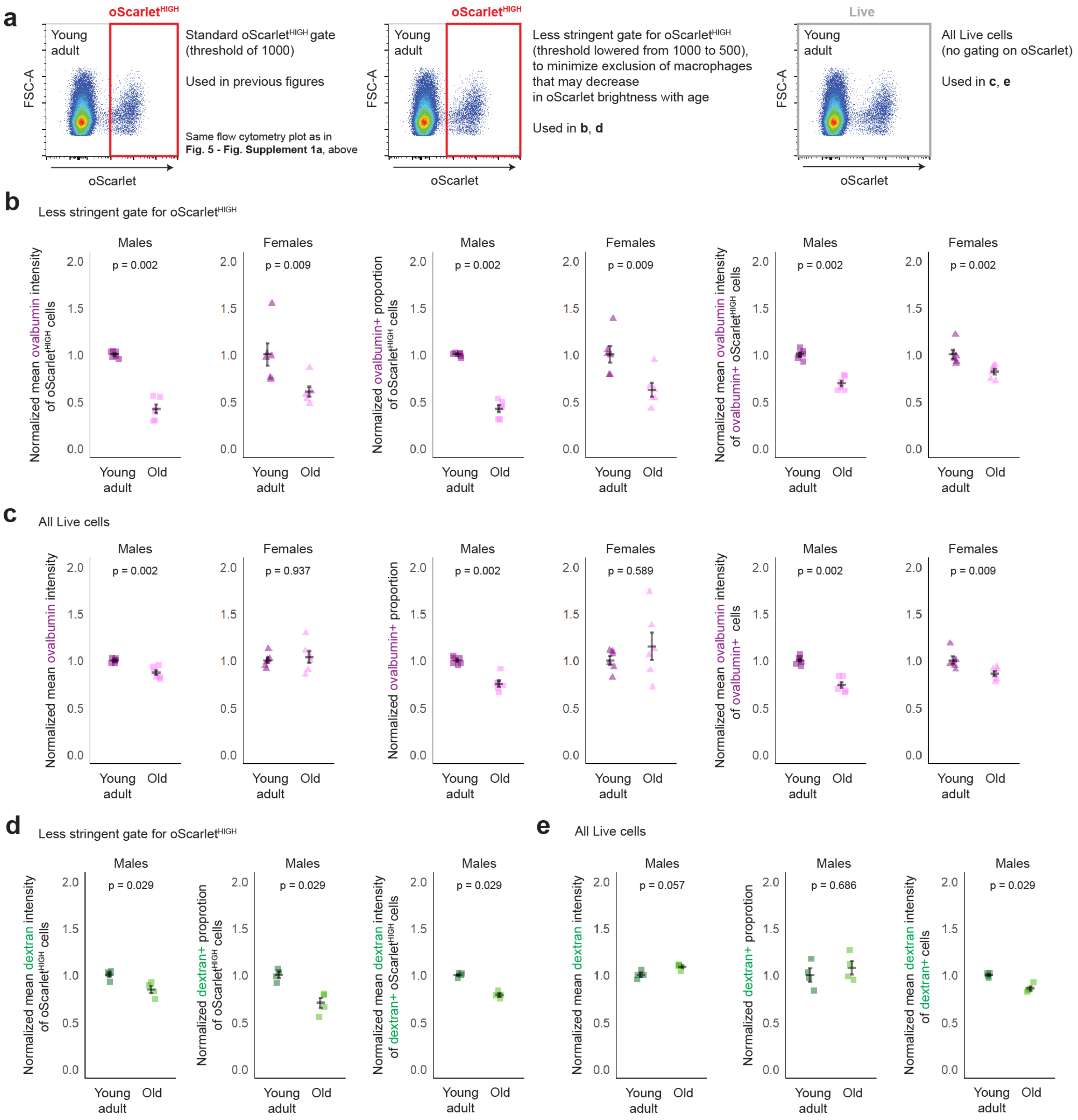

761

bioRxiv preprint doi: https://doi.org/10.64898/2026.04.28.721434; this version posted April 29, 2026. The copyright holder for this preprint (which was not certified by peer review) is the author/funder, who has granted bioRxiv a license to display the preprint in perpetuity. It is made available under aCC-BY 4.0 International license.

Figure 5 – Figure Supplement 2

a, Same flow cytometry plot as in **5S1a** (above), illustrating alternative gating schemes used in subsequent panels.

b, Quantifications of mean ovalbumin fluorescence intensity, proportion of ovalbumin+ cells, and mean ovalbumin fluorescence intensity of ovalbumin+ cells among young adult and old oScarlet^{HIGH} cells (same experiments as in **5a**) with a less stringent oScarlet^{HIGH} gate (lowered from 1000 to 500). Each square (male) or triangle (female) represents one fish. Mean +/- standard error of mean; p-values, unpaired Wilcoxon rank-sum test at per-animal level.

c, Quantifications of mean ovalbumin fluorescence intensity, proportion of ovalbumin+ cells, and mean ovalbumin fluorescence intensity of ovalbumin+ cells among all young adult and old cells (same experiments as in **5a**, no gating for oScarlet). Each square (male) or triangle (female) represents one fish. Mean +/- standard error of mean; p-values, unpaired Wilcoxon rank-sum test at per-animal level.

d, Quantifications of mean dextran fluorescence intensity, proportion of dextran+ cells, and mean dextran fluorescence intensity of dextran+ cells among young adult and old oScarlet^{HIGH} cells (same experiment as in **5e**) with a less stringent oScarlet^{HIGH} gate (lowered from 1000 to 500). Each square represents one fish. Mean +/- standard error of mean; p-values, unpaired Wilcoxon rank-sum test at per-animal level.

e, Quantifications of mean dextran fluorescence intensity, proportion of dextran+ cells, and mean dextran fluorescence intensity of dextran+ cells among all young adult and old Live cells (same experiment as in **5e**, no gating for oScarlet). Each square represents one fish. Mean +/- standard error of mean; p-values, unpaired Wilcoxon rank-sum test at per-animal level.

bioRxiv preprint doi: https://doi.org/10.64898/2026.04.28.721434; this version posted April 29, 2026. The copyright holder for this preprint (which was not certified by peer review) is the author/funder, who has granted bioRxiv a license to display the preprint in perpetuity. It is made available under aCC-BY 4.0 International license.

Supplementary Tables

Supplementary Table 1. Animals used in this study.

Supplementary Table 2. Nucleic acid sequences used in this study.

Supplementary Table 3. Antibodies used in this study.

Supplementary Table 4. Images taken in this study.

Supplementary Table 5. Gene names conversion table used to identify homologs across species in this study.

Supplementary Table 6. Quality control metrics for Seurat objects generated from raw reads of killifish single-cell RNA sequencing data in this study.

Supplementary Table 7. Cluster marker genes based on all young adult cells from August 2025 single-cell RNA sequencing experiment.

Supplementary Table 8. Cell type marker genes based on all young adult cells from August 2025 single-cell RNA sequencing experiment.

Supplementary Table 9. Differentially expressed genes between young adult and old oScarlet^{HIGH} cells from August 2025 single-cell RNA sequencing experiment.

avg_log2FC > 0 indicates enrichment in old oScarlet^{HIGH} cells. avg_log2FC < 0 indicates enrichment in young adult oScarlet^{HIGH} cells. pct. 1 corresponds to the percentage of old oScarlet^{HIGH} cells with detectable expression of the gene. pct. 2 corresponds to the percentage of young adult oScarlet^{HIGH} cells with detectable expression of the gene.

Supplementary Table 10. Gene Ontology: Biological Process (2025) terms upregulated in young adult oScarlet^{HIGH} cells from August 2025 single-cell RNA sequencing experiment.

Supplementary Table 11. Gene Ontology: Biological Process (2025) terms upregulated in old oScarlet^{HIGH} cells from August 2025 single-cell RNA sequencing experiment.

Supplementary Table 12. Differentially expressed genes between old oScarlet^{HIGH} and old wildtype macrophages from October 2024 single-cell RNA sequencing experiment.

avg_log2FC > 0 indicates enrichment in old oScarlet^{HIGH} macrophages. avg_log2FC < 0 indicates enrichment in old wildtype macrophages. pct. 1 corresponds to the percentage of old oScarlet^{HIGH} macrophages with detectable expression of the gene. pct. 2 corresponds to the percentage of old wildtype macrophages with detectable expression of the gene.

Supplementary Table 13. Gene Ontology: Biological Process (2025) terms upregulated in old oScarlet^{HIGH} macrophages from October 2024 single-cell RNA sequencing experiment.

Supplementary Table 14. Gene Ontology: Biological Process (2025) terms upregulated in old wildtype macrophages from October 2024 single-cell RNA sequencing experiment.

bioRxiv preprint doi: https://doi.org/10.64898/2026.04.28.721434; this version posted April 29, 2026. The copyright holder for this preprint (which was not certified by peer review) is the author/funder, who has granted bioRxiv a license to display the preprint in perpetuity. It is made available under aCC-BY 4.0 International license.

Source Data Tables

Source Data Table 1. Corresponding to **Fig. 3a**.

Source Data Table 2. Corresponding to **Fig. 3 – Supplementary Fig. 1a**.

Source Data Table 3. Corresponding to **Fig. 3 – Supplementary Fig. 1b**.

Source Data Table 4. Supporting information for analysis in **Fig. 3b**.

Source Data Table 5. Supporting information for analysis in **Fig. 3b**.

Source Data Table 6. Corresponding to **Fig. 3b**.

Source Data Table 7. Corresponding to **Fig. 5a**, **Fig. 5c-d**, and **Fig. 5 – Fig. Supplement 1c-d**.

Source Data Table 8. Corresponding to **Fig. 5 – Fig. Supplement 2b**.

Source Data Table 9. Corresponding to **Fig. 5 – Fig. Supplement 2c**.

For this analysis, the threshold for oScarlet^{HIGH} was set to 0.

Source Data Table 10. Corresponding to **Fig. 5e-f** and **Fig. 5 – Fig. Supplement 1e-f**.

Source Data Table 11. Corresponding to **Fig. 5 – Fig. Supplement 2d**.

Source Data Table 12. Corresponding to **Fig. 5 – Fig. Supplement 2e**.

For this analysis, the threshold for oScarlet^{HIGH} was set to 0.

Additional Supplements

Supplement 1. SnapGene map file of expected SP-oScarlet insertion.

Supplement 2. Compensation matrix from first *ex vivo* ovalbumin engulfment experiment.

Supplement 3. Compensation matrix from second *ex vivo* ovalbumin engulfment experiment.

Supplement 4. Compensation matrix from *ex vivo* dextran engulfment experiment.
